# The Readiness of Postgraduate Health Sciences Students for Interprofessional Education in Iran

**DOI:** 10.5539/gjhs.v7n4p190

**Published:** 2014-12-31

**Authors:** Zohreh Vafadar, Zohreh Vanaki, Abbas Ebadi

**Affiliations:** 1 Health Research Management center, Nursing Faculty, Baqiyatallah University of Medical Sciences, Tehran, IR Iran; 2 Department of Nursing, Faculty of Medical Sciences, Tarbiat Modares University, Tehran, IR Iran; 3 Behavioral Sciences Research Center (BSRC), Nursing Faculty, Baqiyatallah University of Medical Sciences, Tehran, IR Iran

**Keywords:** interprofessional education, health sciences, postgraduate students, Iranian University of Medical Sciences, RIPLS

## Abstract

**Aim:**

Interprofessional education has been recognized as an effective educational approach towards enabling students to provide comprehensive and safe team care for promotion of health outcomes of patients. This study was conducted in order to assess the readiness of postgraduate health science students for interprofessional education/learning, as well as identify barriers to the implementation of such an approach in Iran from the students’ point of view.

**Methods:**

This was a cross–sectional and descriptive-analytical study conducted in 2013 on 500 postgraduate students in three main professional groups: medical, nursing and other allied health professions across a number of Iranian Universities using the convenience sampling method. Quantitative Data were collected through self-administering the Readiness for InterProfessional Learning Scale (RIPLS) questionnaire with acceptable internal consistency (α = 0.86). The data were analyzed by SPSS18. Qualitative data were gathered by an open–ended questionnaire and analyzed by qualitative content analysis method.

**Results:**

The mean score of the students’ readiness (M=80, SD=8.6) was higher than the average score on the Scale (47.5). In comparison between groups, there was no statistically significant difference between groups in their readiness (p>0.05). Also four main categories were identified as barriers to implementation of interprofessional education from the students’ point of view; the categories were an inordinately profession-oriented, individualistic culture, style of management and weak evidence.

**Conclusion:**

An acceptable degree of readiness and a generally favorable attitude among students towards interprofessional education show that there are appropriate attitudinal and motivational backgrounds for implementation of interprofessional education, but it is necessary to remove the barriers by long-term strategic planning and advancing of interprofessional education in order to address health challenges.

## 1. Introduction

Human healthcare needs are so complex today that they cannot be met by one specialty or a particular professional group alone. A team of healthcare professionals from different disciplines maintaining effective, continuous, and constructive communication among its members is required to meet the increasing needs of humans in a complex environment affected by various elements (Barrett, Greenwood, & Ross, 2003; Hammick, Freeth, Koppel, Reeves, & Barr, 2007; Hean & Dickinson, 2005; Horsburgh, Merry, & Seddon, 2005; Momeni, Ashourion, Abdolmaleki, Irajpour, & Naseri, 2011; Sargeant, Loney, & Murphy, 2008). Accordingly, health science educational organizations should plan to train a new generation of health workers who are capable of playing a role as members of healthcare teams with productive, effective, flexible and adaptable relationships, thus improving the health of present and future generations (D’Amour & Oandasan, 2005; Oandasan & Reeves, 2005; Parsell & Bligh, 1999; Reeves, Freeth, McCrorie, & Perry, 2002).

The World Health Organization (1973) has warned about the inability of health sciences graduates to provide care as members of healthcare teams and the negative consequences of such inefficiency. At a meeting in Alma-Ata, Kazakhstan (1978), WHO researchers first introduced multi-professional education as an effective method for advancing teamwork and improving the quality and security of care. Exactly a decade later (1988), the same international organization published a report entitled “Learning Together to Work Together for Health” recommending interprofessional education (IPE) as a means of addressing the health issues facing those who worked toward the realization of the motto “Health for All by 2000”. The WHO report placed an emphasis on the implementation of its professed goals through educational institutes (Buring et al., 2009; Norman, 2005; Solomon, Thompson, & Tilden, 2009; World Health Organization (WHO), 1998).

Interprofessional education emphasizes learning with, from and about each other in order to achieve a mutual understanding, common goals and collective responsibility among all students in health sciences (Barr, Freeth, Hammick, Koppel, & Reeves, 2006; Faresjo, 2006; Margalit, Thompson, Visovsky, & Geske 2009). It prepares and enables students to deliver comprehensive, safe and collaborative team care and thus improve patients’ health outcomes (Reeves & Sully, 2007; Reeves et al., 2010; Vafadar, Vanaki, & Ebadi, 2014).

The most important [intended, anticipated?] end results of interprofessional education include enhancing the quality of care, effective teamwork, satisfaction in patients and care providers, lower cost and shorter periods of hospitalization, minimization of medical errors and duplication as well as misunderstandings, hostilities, defensive behaviors, stereotyping and conflicts (Barr, Koppel, Reeves, Hammick, & Freeth, 2005; Lapkin, Levett-Jones, & Gilligan, 2013; Thistlethwaite & Moran, 2010).

Today, more than 30 years into interprofessional education, with the trend growing internationally, especially in developed countries, many of the goals have been implemented and research and knowledge about it are enlarging and expanding. However, despite the general global enthusiasm voiced for interprofessional education, it has not yet been formally incorporated in health science education in Iran (Irajpour, Barr, Abedi, Salehi, & Changiz, 2010). Perhaps the reason is a lack of accurate cognition of this educational approach in Iran. Also studies on how to incorporate it to Iranian health systems have been few and far between; neither has a suitable interprofessional education model for Iranian health systems yet been designed (Garrusi & Garrusi, 2012). This is while our society faces major challenges in the areas of quality and security of patient care and suffers from a poor culture of teamwork in the area of health care (Asadi & Akbari, 2008; Vaismoradi, Salsali, Esmailpour, & Cherachi, 2011).

Considering the undeniable necessity of training a new generation of healthcare workers, WHO (2010) has provided a framework for the implementation of interprofessional education with changes introduced in some of the main areas including health education management systems and educational and clinical environments. WHO has also called on different countries in the six regions of the World Health Organization to help with the advancement of interprofessional education emphasizing that the time has come for expanding and developing interprofessional education as a new agenda for dealing with the challenges of healthcare services for patients, and campaigning to highlight the inevitable need for a culture change in healthcare organizations toward interprofessional education and collaboration (WHO, 2010; Jean Yan, Gilbert, & Hoffman, 2007; Jean, Yan, Gilbert, & Hoffman, 2009).

According to Parsell & Bligh, the successful implementation of interprofessional education depends on the creation of structural, institutional and attitudinal changes in health education organizations, and that attitudinal changes are more infrastructural and can induce structural and institutional changes (Parsell & Bligh, 1999). Success of an educational program, irrespective of its content and structure, depends on the need for that program being felt, preparation, and the attitude of the students involved and their understanding of the importance of the program (Coster et al., 2008; Lauffs et al., 2008). Furthermore, the attitude of the students reflects their cognition of the society’s realities, also the impacts of obvious and hidden curriculum (Curran, Sharpe, Flynn, & Button, 2010; Curran, Sharpe, Forristall, & Flynn, 2008).

One of the concerns of Iranian policymakers in the field of health education is whether interprofessional education is applicable or transferable to Iranian society, whether conditions, capacities and facilities to apply it exist, and if our academic community will be receptive to this approach. Without doubt one of the important conditions is propensity and readiness, also motivation and positive attitude among students, not only because the main customers of educational organizations are students, but also because the purpose of education is growth, learning and changes in cognition, perception and behaviors of students (Hammick, Olckers, & Campion-Smith, 2009; Hean, Craddock, & O’Halloran 2009). Moreover, in Iran little is known about interprofessional education or of the attitudes of healthcare students towards it, so this study is the first step in assessing the background of implementation of interprofessional education in Iran. Our study was conducted with the overall aim of assessing the readiness of Iranian postgraduate students of health sciences for interprofessional education/learning as well as the barriers to its implementation from a student’s viewpoint.

## 2. Methods

This was a cross–sectional and descriptive-analytical study conducted in 2013 on 500 postgraduate health sciences students from different disciplines in masters and PhD degrees as well as residents from a variety of specialty programs. Three key governmental agencies are responsible for health sciences education in Iran, namely the Ministry of Health and Medical Education, Ministry of Science, Research and Technology and the General Staff of the Armed Forces. So we selected the largest universities under the supervision of these agencies for this study. Tehran University of Medical Sciences, representing the Ministry of Health and Medical Education, was founded in 1934. It currently has 11 faculties with 19,000 students as well as 18 hospitals with over 3,000 beds and 70 research centers. Tarbiat Modarres University, a representative of the Ministry of Science, Research and Technology, was founded in 1980; it boasts 14 faculties with over 8,000 postgraduate students in a variety of disciplines. Finally, Baqiyatallah University of Medical Sciences which is affiliated to the Armed Forces was founded in 1995 and operates 5 faculties with 1,000 students as well as 16 research centers and 3 hospitals with over 2,000 beds. Due to the large sample size and the vast distribution of subjects, convenience sampling method was used. The instrument for gathering data was a questionnaire consisting of two main parts. The first part covered demographic information such as sex, age, discipline and degree, history of educational and clinical performance and previous experience of attending interprofessional education programs. The second part was “Readiness for Interprofessional Learning Scale” (RIPLS), designed and developed by Parsell & Bligh at Liverpool University for measuring the readiness of health sciences students for Interprofessional Learning/education (Parsell & Bligh, 1999). At the end of the second part, an open question was added asking students about barriers to the implementation of interprofessional education in Iran. RIPLS consists of 19 items in three subscales comprising (i) teamwork and collaboration subscale consisting of 9 items, (ii) professional identity subscale consisting of 7 items, and (iii) roles and responsibility subscale consisting of 3 items, based on the five-point Likert rating scale, (highly disagree=1) to (highly agree=5). So the total score of samples can vary between 19 and 95. In this study, the mean score of scale (47.5) was considered as an average limit of the readiness of students. Internal consistency of RIPLS reported by Parsell & Bligh is (0.9) (Parsell & Bligh, 1999). This scale was translated into Persian and validated by Irajpour in Iran (Irajpour & Alavi, 2011). We used the translated questionnaire prepared by Irajpour. Reliability was confirmed by test-retest (r=0.89, P=0.001), also the internal consistency of the questionnaire was approved by Cronbach’s alpha (α = 0.86). Gathering data was done with direct reference by the researcher to the samples, with the informed consent of the participants, and through delivering the questionnaire to the participants and returning it in compliance with the desire of the students. The definition of interprofessional education, as provided by the Centre for the Advancement of Interprofessional Education (CAIPE, 1997) (‘‘occasions when two or more professions learn with, from and about each other to improve collaboration and the quality of care’’), which has international acceptability, was included in the first page of the questionnaire to ensure that all the students taking part had the same understanding of the concept. Quantitative data were managed and analyzed using SPSS18 as well as descriptive and analytic statistical tests. Qualitative data were analyzed using the qualitative content analysis method. Answers to the open question were transferred to a file exactly. After that, meaningful units and codes were identified. Next, codes were compared with each other and were categorized based on their similarities and differences. Ultimately categories were integrated and consolidated to determine main categories that were indicative of barriers to the implementation of interprofessional education from the students’ point of view.

Considering the scattering of the samples and the variety of the disciplines, for the purpose of analyzing the data, the samples were divided into three main professional groups comprising nursing, medical and other allied health professions such as midwifery, nutritionist, clinical psychology, pharmacy, occupational therapy, physical therapy, laboratory scientist, rehabilitation, social worker, reproductive health, etc., as well as basic medical sciences such as immunology, microbiology, physiology, anatomy and so on. The ethics committee of the Health Management Research Center at Baqiyatallah University of Medical Sciences approved the study.

## 3. Results

Out of 500 distributed questionnaires 420 (84%) were returned: 38% by participants in the medical group, 22% by the nursing group, and 40% by other professions, with the mean age standing at 32±5 in the range of 23 to 50 years old and with 55% of these being male, and 48% studying for a masters degree while 52% worked on a PhD degree or were residents. None of the participants had a previous experience of interprofessional education. However, a majority (68%) had the experience of side-by-side or parallel education with other professions. The total mean score of readiness of students was (M=80.8, SD=8.6) in the range of at least 51 to maximum 94. This score was higher than the average score of scale (47.5). As well as in groups, the mean in 3 subscales was higher than the average score of each subscale. For comparison between groups we used the ANOVA test. This parametric statistical test showed that there were not statistically significant differences between groups in their degree of readiness for interprofessional education (P>0.05). However, despite the lack of significant difference between groups, the mean score of the total scale as well as subscales in the nursing group was higher than in other groups (Table 1).

**Table 1:** Comparison of mean and standard deviation of scale and subscales among professional groups

Variable → Group ↓	Total scale M(SD)	Collaboration and Teamwork subscale M(SD)	Professional identity Subscale M(SD)	Roles and responsibility Subscale M(SD)
Nursing	83.3(8.1)	41.2(3.4)	30.1(2.5)	12.0(2.2)
Medical	79.3(7.8)	39.6(3.7)	28.2(3)	11.2(1.1)
Other professions	80.1(7.6)	40.0(2.8)	28.5(1.8)	11.2(1.8)
Total	80.8(8.6)	40.2(4.6)	29.0(3.8)	11.5(1.8)
ANOVA test	F =0.83, df = 2	F =0.81, df = 2	F =0.62, df = 2	F =0.06, df = 2
	P = 0.43	P = 0.44	P = 0.78	P = 0.93

Note. M= Mean score, SD= standard deviation score.

In qualitative data with content analysis method, 4 main categories were identified as barriers to the implementation of interprofessional education in Iran from the point of view of students. These categories consisted of 1- Inordinately profession-oriented, 2- Individualistic culture, 3- Style of management and 4-Weak evidence. Each category includes many subcategories (Table 2).

**Table 2:** Main categories and subcategories of the barriers to the implementation of interprofessional education

Main Categories	Subcategories
Inordinately profession-oriented	Professional prejudice, professional rivalry, power hierarchy
	Interprofessional power imbalance, physicians predominance
Individualistic culture	Poor communication, highlighted personal performance
Style of management	Centralized management, vertical organizations, segregated faculties
Weak evidence	Inadequate research, lack of awareness about interprofessional education

1- Inordinately profession-oriented: a long tradition of uniprofessional education in health sciences in Iran, as well as lack of opportunities for interprofessional interactions has caused the creation of profession-orientation and formation of firm boundaries separating the professions. Under these circumstances, bridging the existing gap between professions is difficult or impossible. On the other hand, the history of professional evolution has caused predominance of the medical profession over other allied health professions resulting in the formation of a power hierarchy, a perceived sense of inequity in social and professional status and injustice impeditive to creating an environment based on mutual understanding and respect as well as collaboration.

The following are quotations from written answers:

*“Unfortunately there is a tribal perspective held among the professions, so much so that any crossing over the boundaries is perceived as an act of aggression.”*

*“The culture of predominance of physicians is a hindrance to proper attention to other professions and to the importance of their roles.”*

2- Individualistic culture: an individualistic culture is dominant in the Iranian society. In such establishments as health and educational organizations often the rules and regulations are enforced with an emphasis on individual activities. Evaluations are based on personal performance not teamwork. Individuals rather than teams are praised or punished. These circumstances weaken interpersonal relationships and give rise to rivalry and hostility among professions. These are quotations from written answers:

*“Teamwork is really neglected. Unfortunately we have an individualistic culture, so it is really hard for people to see themselves as members of a team. No individual is ever really expected to work as a member of a team.”*

3- Style of management: centrality of management and decision-making at the top of the organizational hierarchy in health systems has been a cause of disinterest in participation of professional groups in initiatives and of their resistance to changes. Vertical organizations lengthen and rather constrict communication channels and so reduce constructive relationships. Also management of each faculty is conducted in isolation and with a propensity for segregation of the faculty from other departments, leaving little opportunity for interaction between members of different faculties. The following are quotations from written answers:

*“Our faculties are completely detached one from the other. Even if you wish to coordinate a program between two departments, it is very hard. So the implementation of a common education will be impossible”*.

4- Weak evidence: as yet interprofessional education is unknown to many universities’ officials. Moreover, little research has been done in the field of health education issues or about their correlation to patients’ health condition. As a result of inadequate and weak evidence, policymakers in health sciences education tend to disregard the importance and necessity of interprofessional education and teamwork. The following are quotations from written answers:

*“The need for interprofessional education in health education systems is not felt by policymakers or by faculty members. Lack of effective evidence indicative of the correlation between teamwork and health outcomes causes most people to imagine that working well on one’s own must be enough”*.

## 4. Discussion

This study was conducted to assess the readiness of Iranian postgraduate students in health science courses for interprofessional education/learning, and to determine the barriers to the implementation of such an approach from a student’s point of view. The results showed that readiness among students was high, acceptable and even considerable. There was no statistically significant difference between professional groups in their readiness. This finding suggests that there is a common understanding, consensus and sense of need among students for interprofessional education/learning. Therefore, there are appropriate attitudinal and motivational backgrounds for using interprofessional education in Iranian health education systems. On the other hand, the high readiness of the students reflects the impact of perceived social realities and students’ awareness of educational and clinical challenges and of the negative results of uniprofessional education.

Among professional groups, the mean score of the nursing group stood above that of other groups, a finding attributable to the fact that nurses constitute the largest professional group in the health system and are located in the frontline of given care, so that they experience more challenges in interprofessional relationships and team care, interprofessional collaboration and effective teamwork being essential to the successful outcome of their contribution. The present study is consistent with many studies within and outside the country.

Irajpour and his colleagues at Isfahan University of Medical Sciences assessed the readiness of health science students for interprofessional education/learning. Their findings showed that there was high acceptance and a positive attitude to interprofessional education among students. It was shown by Irajpour et al that there was not a statistically significant relationship between demographic variables and the readiness of students (Irajpour & Alavi, 2011).

In the study conducted by Rose and his colleagues on the assessment of the attitude and readiness of students in medicine, nursing, occupational therapy, and physical therapy toward interprofessional education, the findings suggested that the readiness of all students was above the average of scale, but that nursing and occupational therapy groups were significantly readier than other groups (Rose et al., 2009).

Also, the study by Aziz and colleagues in Malaysia for assessing the attitude of medical, nursing and pharmacy students to interprofessional learning suggested that the readiness of nursing and pharmacy students was significantly greater than that of medical students, particularly with regard to collaboration and teamwork (Aziz, Teck, & Yen Yen, 2011).

In the study by Hertweck on the comparison between the attitude of physician assistant (PA) students and students from other health professions to interprofessional learning using the Readiness for Interprofessional Learning Scale (RIPLS), PA students showed significantly less readiness than other health profession students towards interprofessional collaboration/learning. Also, gender and experiential differences influenced readiness for interprofessional learning (Hertweck et al., 2012).

El-Zubeir from the United Arab Emirates (UAE) University examined attitudes and readiness of senior medical and nursing students toward interprofessional education and concluded that both groups believed that there are potential academic and clinical benefits in interprofessional learning. Nevertheless, analysis of variance indicated significant differences between the two groups, and nursing students had more readiness than medical students especially in collaboration and teamwork subscale (El-Zubeir, Rizk, & Al-Khalil, 2006).

Garrusi and Garrusi conducted a study to assess stereotyping by medical and nursing students and the possibility of implementation of interprofessional education between these two large professional groups in the Iranian health education system. The findings showed that although the two groups were in agreement on the implementation of interprofessional education, medical students tended to feel superior to the stereotype of the other group. The authors stated that long-term uniprofessional education given to these groups had strengthened their negative stereotypes of each other, and that a sense of superiority among medical students was a main obstacle to providing collaborative team care in clinical situations (Garrusi & Garrusi, 2012).

The present study showed that none of the students had an experience of interprofessional education, but that the majority of them had the experience of parallel or side-by-side education. According to the social contact theory of Alport (1954), one of the negative outcomes of side-by-side education without purposeful interaction and common goals is the reinforcement of negative stereotypes of professional groups (Bridges & Tomkowiak 2010; Hean & Dickinson, 2005). According to studies, students enter the educational environment with preconceived stereotypes of their profession and other professions which are usually rooted in the culture of the society (Ateah et al., 2011; Clark, Cott, & Drinka, 2007; Hall, 2005; Horsburgh, Perkins, Coyle, & Degeling, 2006). In side-by-side education often there are not planned and purposeful interactions between learners; in these circumstances negative stereotypes are strengthened perhaps causing internal pain and infuriation in students. Continuation of these circumstances will lead to negative reciprocity and will disrupt effective relationships, trust and collaboration. Therefore the most damage would be done to patients/clients of health care (Herbert, 2005; Irvine, Kerridge, McPhee, & Freeman, 2002). Unfortunately side-by-side education in many educational environments is encouraged for economic reasons.

The findings in the qualitative part of our study are indicative of major barriers to the implementation of interprofessional education in Iran, as understood by students. These barriers reinforce the necessity of implementation of interprofessional education in Iran, since many of the barriers are in fact the undesirable fruits of long-term uniprofessional education in the country such as instituted profession-oriented attitude, inflexible and rigid boundaries between professions, poor communication, emphasis on individual performance, disrupted interprofessional respect and trust, rivalry and defensive behaviors.

In her study, Mehrabi showed that the professionalization process in the course of an education is the most influential factor in future interprofessional relationships. The current system of uniprofessional education has reinforced the negative stereotypes and physician domination and has created power tiers, a manifestation of upper-hand-lower-hand pattern and a fading of participation–collaboration pattern between these groups. Mehrabi emphasized the formation of a participation–collaboration pattern through interprofessional education, because it is the best pattern for providing effective care; she also recommended changes in professionalization process through promotion of structured interprofessional interactions, respecting roles and the dignity of all professions, alignment of goals and efforts, a balance of power and participation in decision-making (Mehrabi, 2012).

Decision-making at the top levels of organizations and notifying policy decisions down the lower levels have caused the process of applying new educational initiatives to be lengthened. Moreover, the existence of conditions such as rivalry, poor communication, and inflexible boundaries can interfere with the application of interprofessional education measures causing a deterioration of such efforts.

Judging by the acceptable readiness and the favorable attitude of students toward interprofessional education added to the aforementioned barriers, it is imperative that in the case of Iran interprofessional education commences in small steps followed by a step-by-step promotion of interprofessional education and overcoming of the obstacles. The existing barriers between professions may, for instance, be removed in small measure through the formation of small interprofessional groups among close professions on the basis of common interests. The challenges and their outcomes should be assessed for future development. Furthermore, the results should be disseminated to attract new efforts as well as the attention of officials and policymakers. More studies are needed before an interprofessional education model can be designed that will be appropriate to the Iranian culture and which will tackle the existent barriers while drawing on the capabilities and existing resources to encourage faculty members’ initiatives, development of curricula and improvement of education management systems.

## 5. Conclusion

This study was the first step in assessing a possible background for the implementation of interprofessional education in Iran and designing a suitable model for it. The aim of this study was also to inform healthcare policymakers of the benefits of interprofessional education for medical and healthcare students. Acceptable readiness and favorable attitude of students toward interprofessional education, found through conducting a survey, indicates that there are appropriate attitudinal and motivational backgrounds. However, it is necessary to remove barriers set against such an initiative through long-term strategic planning and advancing interprofessional education in order to address existing and future healthcare challenges. Of course further studies are needed to investigate other factors that may have a significant effect on the successful implementation of interprofessional education. The researchers hope that this study will contribute to the promotion of health sciences education and, through that, improvements in the health of the community.
